# CONVENING A NATIONAL CONSENSUS CONFERENCE TO PROMOTE DEMENTIA CAREGIVERS' ENGAGEMENT AND SUPPORT IN CARE DELIVERY

**DOI:** 10.1093/geroni/igac059.285

**Published:** 2022-12-20

**Authors:** Joan Griffin

**Affiliations:** Mayo Clinic, Rochester, Minnesota, United States

## Abstract

The goal of the 2021 Conference on Engaging Family and Other Unpaid Caregivers of Persons with Dementia in Healthcare Delivery, funded by the NIA, was to establish a policy- and practice-aligned research agenda for enhancing dementia caregivers’ engagement and support in health and long-term care settings. Using the CITRA model as a guiding framework, Dr. Griffin will discuss important planning decisions leading up to the event, including the formation of a Steering Committee, selection of expert panelists and conference participants, and preparation of the non-technical background report. Her presentation will also describe the format of the consensus conference itself, focusing on the structure of the panel presentations and consensus activities, including small group breakout sessions in which conference attendees were charged with generating research recommendations; the process for voting on and prioritizing the identified recommendations; and method for refining and organizing the recommendations into major priority areas.

